# Post-Operative Complications in Living Liver Donors: A Single-Center Experience in China

**DOI:** 10.1371/journal.pone.0135557

**Published:** 2015-08-13

**Authors:** Zhongquan Sun, Zhiyong Yu, Songfeng Yu, Jihao Chen, Jingqiao Wang, Cheng Yang, Mengmeng Jin, Sheng Yan, Mangli Zhang, Min Zhang, Shusen Zheng

**Affiliations:** 1 Division of Hepatobiliary and Pancreatic Surgery, Department of Surgery, First Affiliated Hospital, School of Medicine, Zhejiang University, Hangzhou, Zhejiang Province, China; 2 Key Laboratory of Combined Multi-organ Transplantation, Ministry of Public Health, Hangzhou, Zhejiang Province, China; 3 Key Laboratory of Organ Transplantation, Hangzhou, Zhejiang Province, China; Beckman Research Institute of City of Hope, UNITED STATES

## Abstract

The gap between the growing demand for available organs and the cadaveric organs facilitates the adoption of living donor liver transplantation. We retrospectively identified and evaluated the post-operative complications as per the modified Clavien classification system in 152 living liver donors at at the First Affiliated Hospital, College of Medicine, Zhejiang University between December, 2006 and June, 2014. Post-operative complications were observed in 61 patients (40.1%) in the present study, but no mortality was reported. Complications developed in 58 (40.0%) right, 1 (33.3%) left, and 2 (66.7%) lateral left hepatectomy donors. The prevalence of re-operation was 1.3%. Grade I and II complications were observed in 38 (25.0%) and 11 (7.2%) donors, respectively. Grade IIIa complications developed in 9 (5.9%) donors and only 3 (2.0%) patients reported grade IIIb complications. The most common complication was pleural effusion that occurred in 31 (20.4%) donors. No significant prognostic baseline factor was identified in this study. In conclusion, living donors experienced various complications, which were usually mild and had a good prognosis.

## Introduction

Liver transplantation (LTx) is probably the only treatment strategy in patients with terminal liver diseases. However, despite the tremendously growing demand for available organs and mortality of the waiting list, the amount of grafts available is low. This disparity facilitates a more open acceptance of livers procured from other channels, including Living-Donor Liver Transplantation (LDLT). As an alternative of cadaveric liver transplantation, LDLT was first attempted in 1988 [1] and was successfully performed in 1990 [2]. Several advantages, such as shorter waiting time, no warm ischemia time, and diminished cold ischemia time [3], make LDLT an ideal solution, especially when no other grafts are available and the surgery is urgent.

In spite of all the benefits, the safety of living donor remains controversial. Since the adoption of the right lobe liver for LDLT [4], concerns about a perfectly healthy donor receiving a major hepatectomy have emerged; though the risk is low, but almost definite. According to a recent survey, the average prevalence of mortality and morbidity are 0.2% and 24%, respectively [5]. The current consensus is that the donor age, degree of steatosis, and Remnant Liver Volume (RLV) are the most important predictive factors for donor safety, and the right lobe of liver is the most ideal graft in order to avoid poor recipient outcomes [6].

Hepatic steatosis or Non-Alcoholic Fatty Liver Disease (NAFLD) is very common among the population, with prevalence of up to 45% in Western countries and at least 10% in the Asia-Pacific region [7,8]. Steatotic livers exhibit lower tolerance for major hepatectomy and have impaired regeneration capacity. Previous reports indicated that hepatic steatosis is an independent risk factor and occurs as a result of poor donor outcome, which can lead to mortality [9]. Unfortunately, no universal guidelines regarding the safety of the degree of steatosis have been established. Generally, up to 30% fatty infiltration is the highest limit for acceptance policy. An insufficient size of graft, especially when the body weight of recipient markedly exceeds that of the donor’s, will result in Small-for Size Syndrome (SFSS), even death of recipient [10]. On the other hand, early reports indicated that insufficient RLV would have a significant impact on the post-operative liver function and overall survival of the donors [11,12]. Thus, a rational cut-off of RLV is meaningful for clinical practice. Kubota et al. [13] proposed that 40% remnant liver volume (RLV) is acceptable for tumor-free livers, and Fan et al. [14] further extended the limit to 27% in non-hepatic donors.

In earlier literature, the prevalence of donor morbidity ranged from 0–67% [15–18]. This inconsistency was attributed to the lack of evaluation standard for the post-operative complications. Clavien et al. introduced a classification system of surgical complications in 1992 [19], which was modified to address the living donor situation by Broering et al. in 2004 [20]. The modified Clavien classification system was validated by an international survey [21] and supported for assessing post-operative complications after LDLT at the Vancouver International Forum in 2006 [22]. Therefore, the modified Clavien classification system is considered as the established standard for evaluating the living donor outcomes.

In present study, we identified and evaluated the post-operative complications of 152 living donors as per the modified Clavien classification system.

## Patients and Methods

Of the 219 intended living donors, who had applied for liver donation, transplantion was performed for 152 living donors at the First Affiliated Hospital, College of Medicine, Zhejiang University from December, 2006 to June, 2014. All donors were healthy adults and kin to the recipients. The in-hospital and follow-up data was acquired from the patient records and the China Liver Transplant Registry (www.cltr.org), which was authorized as the only National liver transplantation registry in Mainland China by the Ministry of Health in May 2008. The living donor morbidity was graded according to the modified Clavien classification system, which divided the post-operative complications into five grades. The detailed examples were provided by Yi et al. [23].

### Ethics statement

This study was approved by the Ethics Committee of the First Affiliated Hospital, College of Medicine, Zhejiang University, and all aspects of the procedures have been conducted according to the principles expressed in the Declaration of Helsinki. None of the transplant donors were from a vulnerable population and all donors or next of kin provided written informed consent that was freely given. All data were analyzed anonymously and de-identified prior to analysis.

### Donor evaluation

The living donor should apply for liver donation voluntarily and get no financial benefits in whatever form out of the procedure. Only donations between spouse, direct blood relatives, and collateral relatives within three generations were accepted at our center, and every procedure was approved by Ethics Committee of the First Affiliated Hospital, College of Medicine, Zhejiang University. All donors were adult and had the complete capacity to make their own decision. Detailed information of the potential risks regarding the procedure and the right of refusing the surgery at any phase of the donation would be explained to the donors by a experienced surgeon of our center with or without the presence of the rest family members decided by the donors. Written informed consent would be read and signed by donors after discreet consideration. Donor’s age < 18 or > 60 years was considered as unfit for donation, and thus excluded from the donor pool. The ABO identical donor is the most ideal option; however, ABO compatible is also acceptable. No physical or mental morbidity, which might impact the tolerance and awareness of the procedure, should be found in donors. Routine performance of blood cell count, liver biochemistry tests, and hepatitis serological tests were conducted to validate the preliminary exclusion of the donors with underlying liver diseases. Conventional coagulation examinations and tests for infections, including Human Immunodeficiency Virus (HIV) and Cytomegalovirus (CMV), were performed. Chest X-ray and Electrocardiogram (ECG) were routinely performed to exclude any underlying cardiopulmonary diseases. Biliary system abnormality was examined by Magnetic Resonance Cholangiopancreatography (MRCP) and hepatic vascular system was evaluated by Magnetic Resonance Angiography (MRA). Computed Tomography (CT) scan for volumetric measurement was performed to evaluate graft size and the size of remnant donor liver. Remnant volume of < 30% of the whole liver volume was considered hazardous and > 40% was regarded as optimal safety margin for donors. The course of donation was preceded only under the circumstances of having young and healthy donor, and strongly determined when the estimated remnant liver was < 30% or not. Invasive diagnostic procedures, including hepatic angiography and Endoscopic Retrograde Cholangiopancreatography (ERCP) were performed when non-invasive methods failed to exclude any abnormality. If significant hepatic steatosis was suspected during the abdominal ultrasound scan, liver biopsy was performed with the donor’s consent to assess the degree of fatty infiltration. At last, the donors were assessed by an experienced psychiatrist to rule out any mental disability. Self-rating Anxiety Scale (SAS), Self-rating Depression Scale (SDS), and 36-item Short-Form Health Survey Questionnaire (SF-36) were filled by the donors and documented into our database.

### Operative techniques

A Mercedes Benz incision was routinely applied. Donor’s liver was conventionally dissociated. Intra-operative cholangiography, following a cholecystostomy, was performed to portray the biliary anatomy in all the cases. If no abnormality was found, the surgery was continued by parenchyma transection with Cavitron Ultrasonic Suction and Aspirator (CUSA). Hepatic vascular occlusion was not routinely executed unless absolutely required. The incision line was determined by intra-operative ultrasonography. Veins (> 5 mm) were temporarily clipped with titanium clip and were used for further reconstruction in the recipient. After total dissociation of the right lobe, the right hepatic artery, right portal trunk, and right hepatic vein were interdicted and transected in an orderly manner. The hemorrhage and bile leakage were repaired with 5–0 prolene and 6–0 PDS (polydioxanone), respectively, over the transected surface. Cholangiography was repeated to assess the remaining biliary system at the end of the operation. The weight of the graft was measured at the back table and documented into our database. Intra-peritoneal sub-hepatic tube was placed in all cases.

### Postoperative treatment and follow-up

Donors were transferred to Intensive Care Unit (ICU) for liver transplantation right after the procedure. However, once they were stable, the donors were moved to clinical ward (CW). The total hospital Length of Stay (LOS) was calculated as time admitted to the ICU and the time admitted to the CW. The resection time was calculated from the beginning of dissociation of the liver to the end of the repeated cholangiography. The blood cell count, liver biochemistry tests, hepatic vascular status, and RLV regeneration were evaluated during the hospital stay and follow-up. Donor characteristics, operation-related profile, biochemical parameter values, and post-operative complications were collected and analyzed. All the data pertaining to donors was updated to January, 2015 database.

### Statistical analysis

All continuous variables were presented as mean ± standard deviation and categorical variables were presented with counts and percentages. Continuous variables of several groups were compared by ANOVA or the Kruskal-Wallis H test as appropriate. The chi-square test was used to determine categorical variables. The ordered grades of complications were analyzed by ordinal regression and subgroups of grade III (IIIa and IIIb) served as one group. Pre- and intrao-perative variables which were found to be univariately significant at *P* < 0.1 entered ordinal regression to determine the independent risk factors for postoperative complications. All analysis were performed with the statistical software package (SPSS) 19.0 (SPSS Inc, Chicago, IL, USA). *p* < 0.05 was considered to statistically significant.

## Results

During the study period, 216 voluntary donors applied for liver donation for related recipients. After the initial evaluation, 64 donors were rejected for the procedure due to hepatitis B (12, 18.8%), deterioration or death of the recipients(11, 17.2%), vascular or biliary abnormality (8, 12.5%), and other reasons (Table 1). Therefore, LDLT was performed on 152 donors, 140 male donors (92.1%). The median age of the donors was 23 years (mean 26, range 18–53). Most donors (95.4%) donated the right liver without middle hepatic vein (MHV). Five left livers were donated to children and two were used for dual graft liver transplantation. RLV of donors ranged from 24.0–63.7% (mean 41.3 ± 5.3%, media 41.2%). Four donors with less than 30% RLV underwent the transplantation; however, no mortality occurred in these patients. No post-operative complication was reported in three out of the four donors, whereas only one donor reported mild pleural effusion and right pulmonary atelectasis. The donor was treated conservatively, without any invasive therapy, thus, the complication was established as grade II complication. The blood transfusion during the operation was prudently avoided and only one patient received an allo-transfusion of 1000 mL. Other variables are summarized in Table 2.

**Table 1:** Exclusion criteria for the donors.

Exclusion criteria	n
Ethical considerations	16
Hepatitis B donor	12
Recipient deterioration and death caused by liver failure	11
Donor liver vascular or biliary abnormality	8
High bilirubin	5
Poor graft quality (> 30% macrovesicular steatosis)	4
Infectious disease (syphilis, tuberculosis)	3
ABO blood type incompatibility	3
Coagulation abnormality	1
Donor cardiovascular disease (hypertension, left ventricular hypertrophy)	1

**Table 2:** Pre-operative and operative characteristics of 152 living liver donors.

Variables		
**Donor characteristic**		
**Age**		25.81±7.32
**BMI**		21.85±2.04
**Gender**	Male	140(92.11%)
	Female	12(7.89%)
**ABO blood type**	A	47(30.92%)
	B	30(19.74%)
	AB	3(1.97%)
	O	73(48.03%)
**Liver volume with CT (cm** ^**3**^ **)**		1300.67±186.84
**Right liver volume with CT (cm** ^**3**^ **)**		766.54±125.55
**Operative variables**		
**Graft weight(g)**		630.82±119.88
**Mode of donor hepatectomy**	Right hepatectomy (without MHV)	145(95.39%)
	Left hepatectomy (without MHV)	3(1.97%)
	Left lateral sectionectomy	3(1.97%)
	Left hepatectomy (with MHV)	1(0.66%)
**Remnant liver volume (%)**		41.3 ± 5.3%
**Operative time (min)**		426.84±107.17
**Harvesting graft time (min)**		118.75±57.39
**Blood loss (ml)**		312.17±134.21
**Urine volume (ml)**		1189.58±539.14
**Allotransfusion**		1(0.77%)
**Pre-operative biochemical profile**		
**Albumin (g/L)**		47.96±6.05
**Total bilirubin (μmol/L)**		15.24±6.99
**AST (IU/L)**		21.37±13.21
**ALT (IU/L)**		21.31±18.29
**Blood urea nitrogen (μmol/L)**		4.48±1.24
**Creatinine (μmol/L)**		70.09±16.14
**Alkaline phosphatase (IU/L)**		62.87±18.93
**INR**		1.02±0.08
**Phosphorus (mg/dL)**		1.19±0.19

BMI: Body Mass Index; MHV: Middle Hepatic Vein; AST: Aspartate Aminotransferase; ALT: Alanine Aminotransferase; INR: International Normalized Ratio.

### Post-operative liver function

**Fig 1:**
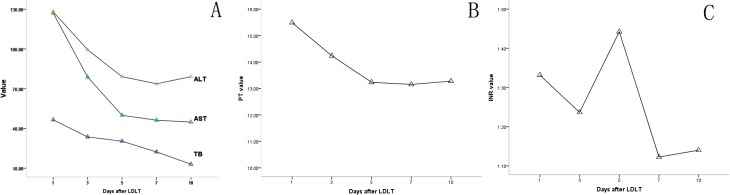
Post-operative biochemical parameter values in living donors after procedure. **A:** TB value returned to normal within the first week after procedure, but AST and ALT recovered after 7 days. **B:** PT value peaked on POD 1 and returned to normal within the first week. **C:** INR descended on the first 3 days after LDLT, but markedly increased and peaked at 4^th^ and 5^th^ day.

Except for International Normalized Ratios (INR), all the post-operative liver functional parameters peaked at the first day after LDLT. INR descended on first 3 days after LDLT, but peaked up at days 4 and 5. The peak values were: total bilirubin (TB): 46.7 ± 27.3 μmol/L; alanine transaminase (ALT): 126.4 ± 58.1 IU/L; aspartate aminotransferase (AST): 127.1 ± 58.1 IU/L; prothrombin time (PT): 15.5 ± 2.3 seconds; INR: 1.44, range 0.94–13.10, respectively. The TB, PT, INR values returned to normal within first week after procedure, but AST and ALT recovered after 7 days (Fig 1).

### Post-operative complications

The post-operative complications were graded according to the modified Clavien classification system (Table 3). In the present study, the post-operative morbidity was identified in 61 patients (40.1%); however, no mortality or grade IV complication was observed. Complications developed in 58 (40.0%) right, 1 (33.3%) left, and 2 (66.7%) lateral left hepatectomy donors. The prevalence of re-operation was 1.3%.

**Table 3:** Complications in liver donors according to the modified Clavien classification system.

Grade		Complications	n
**Grade I**	Single symptom	Pleural effusion (mild)	21
**(n = 38, 25.0%)**		Vomit treated with antiemetic	4
		Wound liquefaction	3
		Seroperitoneum	3
		Low urine volume treated with diuretics	1
		Transient elevation of creatinine	1
	Multiple symptoms	Pleural effusion (mild) and partial atelectasis	1
		Pleural effusion (mild) and subdiaphragmatic effusion (mild)	1
		Pleural effusion (mild) and transient bile leakage	1
		Seroperitoneum and fever treated with symptomatic therapy	1
		Pleural effusion (mild) and sinus bradycardia	1
**Grade II**	Single symptom	Fever treated with antibiotics	2
**(n = 11, 7.2%)**		Bile leakage lasting longer than 1 week	2
		Transfusion of foreign blood plasma	2
		Gastrointestinal tract bleeding treated with hemostatic	1
		Atelectasis treated non-invasive positive pressure ventilation	1
	Multiple symptoms	Dyspnea treated non-invasive positive pressure ventilation and pleural effusion (mild)	1
		Portal thrombosis treated with low molecular heparin and pleural effusion (mild)	1
		Right pulmonary atelectasis and pleural effusion (mild) and subdiaphragmatic effusion (mild)	1
**Grade IIIa**	Single symptom	Pleural effusion (server) using pleurocentesis	4
**(n = 9, 5.9%)**		Abdominal distension treated with gastrointestinal decompression tube	1
		Biloma using percutaneous puncture and drainage	1
	Multiple symptoms	Pneumothorax using closed thoracic drainage and ileus using gastrointestinal decompression tube	1
		Pleural effusion (server) using pleurocentesis and bile leakage using percutaneous puncture and drainage	1
		Pleural effusion (moderate) using pleurocentesis and bile leakage using percutaneous puncture and drainage and fever using antibiotics	1
**Grade IIIb**	Single symptom	Intra-abdominal hemorrhage needing laparotomy	2
**(n = 3, 2.0%)**		Wound debridement and re-suture under intravenous anesthesia	1

The grade of complications was defined according to the highest grade complications in multiple complications.

Thirty-eight patients (25.0%) suffered grade I complications. These patients were treated conservatively and recovered satisfactorily. The most frequently encountered morbidity was mild pleural effusion (n = 25, 16.4%) and is generally automatically absorbed. One donor was identified with high TB in drainage after surgery and therefore, was considered for bile leakage. Upon treatment, the bile leakage reduced within 1 week; classified as grade I complication.

Eleven cases (7.2%) reported grade II complications in this study. Bile leakage was observed in two donors and both were diagnosed on post-operative day (POD) 3 and treated with drainage. One drainage tube was removed on POD 52, while the other tube was carried out for six months. In addition, a grade II donor with portal vein thrombosis was determined by ultrasound on POD 14. The patient was treated with low molecular weight heparin and regularly re-examined after discreet evaluation. After discharge, the patient was administered with warfarin for dissolving thrombus during the follow-up.

Grade III complications were divided into two subgroups based on the involvement of general anesthesia. Nine donors (5.9%) were diagnosed with grade IIIa and 3 (2.0%) with grade IIIb complications. Four kinds of grade IIIa complications were encountered. Pleural effusions in six donors were treated with pleurocan insertion and drainage, and pneumothorax in one patient was treated with chest tube. Bile leakage or bilioma in three subjects was treated with ultrasonography-guided aspiration and pig-tail tube drainage. A 45-year-old male complained recurrent abdominal dull pain after surgery. The patient was diagnosed with incomplete intestinal obstruction, five months following the donation. The symptoms improved after placing a nasogastric decompression tube during the gastroscopy.

Of the three patients undergoing grade IIIb complications, two donors underwent laparotomy due to the intra-abdominal hemorrhage on POD 1. The remaining patient was found to have incisional fat liquefaction on POD 9 and was treated with dressing changing. However, no improvement was observed and the wound ruptured afterwards. Therefore, the patient was subjected to re-suture with intravenous anesthesia on POD 16.

The donor characteristics and pre-operative profile showed no significant difference between the different grades of complications, except INR (Table 4). However, following the ordinal regression, the difference of INR was found not significant (*p* > 0.05), and thus was considered without any significant predictive value for post-operative complications. The overall length of ICU and hospital stay was 4.13 ± 2.27 days and 19.99 ± 8.56 days, respectively. No prolonged ICU stay was identified in the donors with complications. The LOS was significantly longer in patients with grade III complications compared to donors without complications(*p* = 0.046). All the donors resumed their lives during the follow-up.

**Table 4:** Comparison of pre-operative and operative variables between different grades of complications.

Variable		Grade 0	Grade I	Grade II	Grade III^*^	*p*
**n**		91(59.9%)	38(25.0%)	11(7.2%)	12(7.9%)	
**Age**		25.48±6.67	26.45±8.90	25.82±6.82	26.25±7.81	0.918
**Gender (M)**		86(94.5%)	34(89.5%)	9(81.8%)	11(91.7%)	0.442
**ABO blood type**	**A**	27(29.7%)	9(23.7%)	3(27.3%)	8(66.7%)	0.415
	**B**	20(22.0%)	7(18.4%)	2(18.2%)	1(8.3%)	
	**AB**	2(2.2%)	1(2.6%)	0(0.0%)	0(0.0%)	
	**O**	42(46.2%)	21(55.3%)	0(0.0%)	3(25.0%)	
**Mode of donor hepatectomy**	**RH**	87(95.6%)	36(94.7%)	10(90.9%)	12(100.0%)	0.853
	**LH**	2(2.2%)	1(2.6%)	0(0.0%)	0(0.0%)	
	**LLH**	1(1.1%)	1(2.6%)	1(9.1%)	0(0.0%)	
	**LH with MHV**	1(1.1%)	0(0.0%)	0(0.0%)	0(0.0%)	
**BMI**		21.77±1.83	21.94±2.30	21.92±2.42	22.04±2.56	0.957
**Graft weight (g)**		630.33±120.33	634.32±101.19	587.82±179.38	662.83±110.13	0.516
**Operative time (min)**		428.95±110.53	416.16±106.13	399.09±74.99	462.50±109.80	0.475
**Harvesting graft time (min)**		119.72±58.58	116.66±62.67	129.90±55.22	108.75±29.88	0.848
**Blood loss (mL)**		313.19±136.39	302.63±120.78	290.91±115.80	354.17±176.40	0.653
**Urine volume (mL)**		1210.81±575.20	1206.94±554.10	1110.00±387.87	1051.67±301.87	0.763
**Liver volume with CT (cm** ^**3**^ **)**		1303.78±198.36	1285.16±150.60	1321.10±217.15	1308.14±192.96	0.938
**Right liver volume with CT (cm** ^**3**^ **)**		770.66±136.27	744.24±93.74	786.56±137.08	789.558±124.63	0.586
**Remnant liver volume (%)**		41.41±5.61	41.91±4.62	40.27±6.00	39.53±4.87	0.531
**Albumin (g/L)**		48.09±5.98	47.56±6.90	47.95±5.59	48.34±4.41	0.973
**Total bilirubin (μmol/L)**		15.73±7.84	14.21±5.24	15.70±5.58	14.55±6.68	0.716
**AST (IU/L)**		21.58±12.13	22.19±18.03	18.60±6.22	19.75±7.24	0.857
**ALT (IU/L)**		22.54±21.18	20.38±15.08	17.30±7.09	18.83±10.18	0.761
**Blood urea nitrogen (μmol/L)**		4.43±1.11	4.42±1.17	4.82±1.58	4.68±1.90	0.739
**Creatinine (μmol/L)**		70.06±15.73	70.09±17.40	67.70±16.48	72.25±16.50	0.935
**Alkaline phosphatase (IU/L)**		62.38±19.85	64.67±16.35	59.90±19.41	63.50±20.85	0.889
**INR**		1.04±0.08	1.00±0.08	1.00±0.07	1.00±0.08	0.037
**Phosphorus (mg/dL)**		1.18±0.20	1.21±0.18	1.22±0.16	1.15±0.22	0.729
**Length of ICU stay (days)**		3.92±1.96	4.11±2.00	4.64±1.75	5.25±4.56	0.239
**LOS (days)**		18.80±8.00	20.03±6.55	22.82±12.58	26.25±11.55	0.023

Grade IIIa and IIIb complications are combined as one variable.

ALT: Alanine Aminotransferase; AST: Aspartate Aminotransferase; BMI: Body Mass Index; INR: International Normalized Ratio; LH: Left Hepatectomy; LLH: Lateral Left Hepatectomy; LOS: Length of Hospital Stay; MHV: Middle Hepatic Vein; RH: Right Hepatectomy.

## Discussion

Donor safety is the prime priority associated with LDLT procedure. Due to the complexity of the surgery, post-operative complications are generally inevitable. Previous studies have reported the morbidity rate of donors in the range of 0–67% [15–18]. This arresting inconformity was attributed to the extent of donor liver resection and lack of uniform definition of complications. According to Renz et al. [24] centers with more experienced personnel tends to encounter fewer complications, thus we propose that such phenomenon may also be due to the variable experience of different transplantation centers. At our center, the overall prevalence of post-operative complications was 40.1%.

Following the adopting of right hepatectomy, there was a notable augment of the incidence of morbidity. Donors underwent right hepatectomy had smaller remnant liver volume than those with left hepatectomy, thus deteriorated the recovery. Our results showed that right lobe donors suffered complications less frequently than left segment donors (40.0% vs. 42.9%); however it was noticeable that only seven cases of left hepatectomy donors were included in this study, hence the morbidity rate was debatable.

Hashikura et al. analyzed 3565 living donors in Japan, and indicated that the most frequently encountered complications were bile leakage and wound infection [25]. However, bile leakage and wound infection were rare at our center, with prevalence of 2.6% (4/152) and 2.6% (4/152), respectively. This low incidence of bile leakage is mainly due to the routinely performed pre-operative MRCP and intra-operative cholangiography, which provided the complete information of the donor biliary tree as well as the discreetly closure of injured biliary tree after hepatectomy. Only two donors (1.3%) underwent re-operation at our center due to the intra-abdominal hemorrhage. The most common complication in our study was pleural effusion (n = 31, 20.4%); however, most of the reported complications (n = 21, 77.4%) were mild and required no medical intervention.

The worldwide prevalence of donor mortality is 0.2% and 0.16% specifying to the deaths related to surgery [5]. The primary causes of death were sepsis and liver failure [26]. No mortality was observed at our center. The most severe complication was intra-abdominal hemorrhage, which was caused by bleeding of the stump of right branch of portal vein or arteriole near the glisson’s sheath.

Additionally, we analyzed number of risk factors, including donor characteristics and operation-related profiles. Unfortunately, we failed to identify any significant prognostic baseline factors and thus, further investigations are required to validate the study.

The aim of routinely monitoring the changes in laboratory test values was to evaluate the recovery of the donor and to identify complications earlier. In our study, all parameters, including ALT, AST, TB, PT and INR, returned to normal shortly after the procedure. Chan et al. investigated the long-term biological consequences of 29 living liver donors and demonstrated an increase in serum ALT, AST and TB [27]. Though the elevation was statistically significant, the changes remained within normal ranges and had yet uncertain clinical significance.

In conclusion, LDLT is relatively safe for living donors and severe complications are rarely occurred.
